# Boosting the stability of *β*-galactosidase immobilized onto soy-protein isolate-glutaraldehyde-functionalized carrageenan beads

**DOI:** 10.1007/s13205-022-03446-2

**Published:** 2023-01-03

**Authors:** Marwa I. Wahba

**Affiliations:** 1 Department of Chemistry of Natural and Microbial Products, National Research Center, El-Behooth St., Dokki, Giza, Egypt; 2 Centre of Scientific Excellence-Group of Advanced Materials and Nanotechnology, National Research Centre, El-Behooth St., Dokki, Giza, Egypt

**Keywords:** Uncontrolled enzyme-immobilizer interactions, Stability, Soy-protein isolate, *β*-galactosidase, Lactose

## Abstract

Uncontrolled enzyme-immobilizer interactions were evident after immobilizing *β*-galactosidase onto soy-protein isolate-glutaraldehyde-functionalized carrageenan beads. Such interactions triggered shortcomings in the immobilized *β*-galactosidase (iβGL) thermal and storage stabilities. The thermal stability of the iβGL was somewhat lesser than that of the free βGL. Moreover, the iβGL suffered an initial sharp fall-off in its activity after storing it. Thus, approaches were adopted to prevent the occurrence of such uncontrolled enzyme-immobilizer interactions, and accordingly, boost the stability of the iβGL. These approaches involved neutralizing the covalently reactive GA entities via glycine and also altering the functionalizing GA concentrations. Nonetheless, no improvement was recorded in the iβGL thermal stability and this indicated that the uncontrolled enzyme-immobilizer interactions were not mediated via GA. Another approach was then attempted which involved treating the iβGL with lactose. The lactose-treated iβGL (LT-iβGL) presented superior thermal stability as was verified from its smaller *k*_*d*_ and bigger *t*_1/2_ and *D*-values. The LT-iβGL *t*_1/2_ values were 5.60 and 3.53 fold higher than those presented by the free βGL at 62 and 65 °C, respectively. Moreover, the LT- iβGL presented loftier ΔG than did the free βGL. The storage stability of the LT- iβGL was also superior as it offered 100.41% of its commencing activity on its 43rd storage day. Thus, it could be concluded that lactose prevented the uncontrolled enzyme-immobilizer interactions. Finally, advantageous galacto-oligosaccharides (GOS) were prepared via the iβGL. The GOS were then analyzed with mass spectrometry, and it was shown that their degree of polymerization reached up to 7.

## Introduction

Immobilization is pivotal to render the industrial utilization of enzymes more economically feasible. Immobilized enzymes could be re-utilized many times (Bolivar et al. 2022) as they could be simply segregated from their reaction mixtures. The re-utilization of enzymes would reduce the costs of their industrial utilization. Immobilization could also augment enzymes stability. The creation of multipoint covalent attachments betwixt the enzyme and a rigid immobilizer would serve to rigidify the enzyme construction. Such construction rigidification would augment the enzyme stability against disturbing factors, such as solvents and escalated temperatures (Rodrigues et al. 2021). Moreover, linking all multimeric enzymes subunits to an immobilizer (easily achieved in case of dimeric enzymes) would prohibit their dissociation and would prevent their inactivation (Rodrigues et al. 2021).

Furthermore, immobilization could modulate other enzymes traits, such as their selectivity and specificity (Arana-Peña et al. 2021). Enzyme selectivity and specificity modulations would be immensely beneficial if the enzyme exhibited impaired selectivity and specificity toward the intended industrial substrate. Selectivity and specificity modulations would mainly occur in the enzymes that could exert immense conformational alterations without being deactivated. The construction of such enzymes could get distorted secondary to immobilization, and their mobility might also be reduced. Moreover, immobilization could alter the enzymes micro-environments (Garcia-Galan et al. 2011). The aforementioned immobilization induced alterations could eventually lead to drastic alterations in the enzymes features which could include alterations in their selectivity and specificity (Garcia-Galan et al. 2011).

Enzyme immobilization and purification could also be coupled together. For instance, the purification of bulky proteins (> 200 kDa) and their segregation away from smaller proteins (˂100 kDa) could be fulfilled via immobilization. Bulky proteins would cover more of the immobilizer surface, and accordingly, they could establish distanced multi-interactions. Moreover, they would possess more surface residues that are capable of interacting with the immobilizer (Barbosa et al. 2015). Thus, bulky proteins would exhibit enhanced binding capacity, and this would allow for their preferential adsorption onto the immobilizers that possess much diminished surface activation degree. Nonetheless, adsorption accomplished via the immobilizers that possess much diminished surface activation degree could be readily reversed. Thus, it should be associated with covalent immobilization, and this could be fulfilled via utilizing hetero-functional immobilizers. However, the covalent linking should be accomplished only after the initial preferential adsorption to allow for protein purification (Barbosa et al. 2015). Additionally, immobilization could hamper enzyme inhibition. Immobilization induced distortions could increment the *K*_*i*_ to a bigger extent than the *K*_*m*_, and this would diminish the influence of inhibition on the enzymatic reaction. Immobilization onto a solid immobilizer might also cause the blockage of the enzyme inhibition site, and this would totally prohibit the inhibition (Garcia-Galan et al. 2011).

Noteworthy, at the end of the immobilization, the immobilizer should be rendered physically and chemically inert to prohibit the incidence of uncontrolled enzyme-immobilizer interactions (Bolivar et al. 2022). Uncontrolled enzyme-immobilizer interactions might negatively influence the enzyme stability (Barbosa et al. 2014; Bolivar et al. 2022) to the extent that the immobilized enzyme might exhibit lesser stability than its free analogue. For instance, the transaminase, which was immobilized via epoxy grafted resin (ES-105), was less stable than its free analogue as was evident from the loss of the immobilized transaminase activity after 40 min incubation at 57 °C. This instability was regarded to the uncontrolled interactions amid the immobilized transaminase and the ES-105 remaining covalently active epoxy moieties (Jia et al. 2020). Various blocking agents could be utilized to block and neutralize any remaining covalently active moieties (Table 1), and this would render the immobilizer chemically inert. This approach was applied in case of the ES-105 immobilized transaminase. The remaining reactive epoxy moieties were blocked via glycine, and this boosted the thermal stability of the immobilized transaminase (Jia et al. 2020). On another occasion, the vinyl-sulfone moieties, which remained active after finishing lipase immobilization onto divinyl-sulfone-functionalized agarose beads, were blocked via incubation in glycine, ethylenediamine, or imidazole. The blocking boosted the thermal stability of the immobilized-blocked enzyme preparation (Dos Santos et al. 2015). Blocking via incubation in glycine or ethanolamine was also reported to boost the stability of the penicillin G acylase immobilized onto vinyl-sulfone agarose (Da Rocha et al. 2022). Such stability enhancements were probably consequences of inhibiting the uncontrolled enzyme-immobilizer interactions. It was also formerly stated that the remaining reactive epoxy moieties in Eupergit C, Sepabeads EP, and epoxy grafted Purolite^®^A109 (Table 1) were blocked via incubation in glycine (Mihailović et al. 2014; Torres and Batista-Viera 2012). Glycine could also be utilized to block and neutralize the remaining reactive glutaraldehyde (GA) residues. Glycine was previously shown to be capable of neutralizing GA in water (Chen and Roberts 2002).

**Table 1:** Various approaches adopted to render immobilizers chemically inert

Enzyme	Immobilizer	Blocking step	Stabilizing effect imparted by the blocking step	References
*Escherichia coli* penicillin G acylase (PGA)	Divinyl-sulfone-functionalized agarose	The remaining vinyl sulfone moieties were blocked via incubation in ethanolamine or glycine	Improvement in the stability of immobilized PGA	Da Rocha et al. (2022)
*Candida antarctica* lipase B	Divinyl-sulfone-functionalized agarose	The remaining vinyl sulfone moieties were blocked via incubation in ethylenediamine, glycine, ethanolamine, imidazole, or cysteine	Improvement in the thermal stability of immobilized lipase at 60 °C	Dos Santos et al. (2015)
Recombinant *Citrobacter koseri* transaminase	Epoxy-grafted resin (ES-105)	The remaining epoxy moieties were blocked via interaction with glycine	Improvement in the thermal stability of immobilized transaminase at 57 °C	Jia et al. (2020)
*Candida antarctica* lipase B	Epoxy-grafted Purolite^®^A109	The remaining epoxy moieties were blocked via incubation in glycine, phenylalanine, or aspartic acid	Improvement in the thermal stability of immobilized lipase at 65 °C	Mihailović et al. (2014)
*Bacillus circulans* βGL	Eupergit C and Sepabeads EP	The remaining epoxy moieties were blocked via incubation in glycine	Improvement in the thermal stability of iβGL at 40 and 50 °C	Torres and Batista-Viera (2012)

As regards to rendering the immobilizer physically inert, it was formerly mentioned that immobilizers, which offered physically active functionalities, would always exhibit physically active surfaces (Barbosa et al. 2014; Bolivar et al. 2022; Virgen-Ortíz et al. 2017). Thus, if the de-stabilizing uncontrolled enzyme-immobilizer interactions were mediated via physically active functionalities, the approaches adopted to prohibit such destabilizing interactions should not be directed toward neutralizing the immobilizer. Such an immobilizer would always present physically active functionalities. On the other hand, the adopted approaches should be directed toward protecting the enzyme active site and retaining its native configuration. Substrates sturdily bind to the enzyme native configuration, and such sturdy binding stabilize the enzyme presumably owing to conformational tightening (Lejeune et al. 2001). Thus, the addition of substrates to the immobilized enzyme, which suffer from de-stabilizing uncontrolled enzyme-immobilizer interactions, might stabilize it. It should be noted that to the best of our knowledge, no published reports have presented protocols to overcome the de-stabilizing uncontrolled enzyme-immobilizer interactions that were mediated via physically active functionalities. Thus, in the current research, we attempted to present approaches that would overcome both the chemically and the physically mediated de-stabilizing uncontrolled enzyme-immobilizer interactions.

Soy-protein isolate (SPI)-GA-functionalized carrageenan (Car) beads were formerly reported as immobilizers for the industrial enzyme *β*-d-galactosidase (βGL) (Wahba 2022a). These SPI-GA-functionalized Car immobilizers were proficient. They immobilized βGL with 96.45% immobilization efficiency. They also granted an escalated operational stability to the immobilized βGL (iβGL) where 83.37% iβGL relative activity was presented throughout the 12th reaction cycle. During the functionalization of such immobilizers Car beads were coated with an SPI sheath. Afterward, they were allowed to interact with GA (Wahba 2022a). SPI is a protein; thus, it would present cationic and anionic entities whose abundance would be dictated by the adopted pH value. These cationic and anionic entities could mediate ion-exchange with enzymes. Accordingly, the SPI-GA-functionalized Car beads surface would present the physically active SPI residues together with the covalently reactive GA residues. Both of these residues could induce de-stabilizing uncontrolled enzyme-immobilizer interactions. Thus, in the current work, we attempted to prohibit the incidence of such de-stabilizing interactions amid the iβGL and the SPI-GA-functionalized Car beads to preserve the stability of the iβGL.

Galacto-oligosaccharides (GOS) are characteristic for their prebiotic traits as they can promote the propagation of the beneficial microflora within the gastrointestinal tract (Sass and Jördening 2020). Accordingly, GOS are advantageous for humans, and in particular, for babies and infants. Lots of babies consume infant formulas, which are based on bovine milk, as surrogates to the human mothers’ milk, which comprises plenty and diverse oligosaccharides (> 200) (Yin et al. 2017). Accordingly, human milk oligosaccharide substitutes, such as GOS, are integrated within infant formulas. GOS are prepared from lactose following a βGL-mediated transgalactosylation reaction. In a transgalactosylation reaction, the galactosyl residues, which are released following lactose hydrolysis, are transferred to diverse possible acceptors, such as monosaccharides, lactose, or oligosaccharides, and this leads to the formation of GOS (Yin et al. 2017). Thus, in the current work, after resolving the de-stabilizing interactions that occurred amid the iβGL and the SPI-GA-functionalized Car beads, the iβGL was exploited to procure these valuable GOS.

## Materials and methods

### Materials

*Aspergillus oryzae* βGL, Car, and 50% (v/v) GA were attained from Sigma-Aldrich (Germany). SPI with a protein content of 90% was attained from BulkSupplements.com (USA). Commercial glucose estimation kits were acquired from Spinreact, Spain.

### Methods

#### Preparation of the SPI-GA-functionalized Car beads

Car powder was dissolved in distilled water via stirring in a 70 °C water bath. The hot Car solution (2%, w/v) was then extruded through a strait syringe needle into a 3% (w/v) KCl gelling solution. The acquired Car beads were kept in the KCl solution for at least 2 h. Afterward, they were meticulously washed and soaked overnight within 10% (w/w) SPI suspension of pH 5. The beads were then meticulously washed and soaked within GA solution (25%, v/v) for 1 h (Wahba 2022a). Finally, the SPI-GA-functionalized Car beads were washed and put in the fridge in distilled water till loading them with βGL.

#### βGL loading

The powdered βGL enzyme was dissolved in citrate–phosphate buffer (pH 4.6; 0.3 M) and was then mixed with the SPI-GA-functionalized Car beads. Such a mixture was rotated via a roller stirrer for around 18 h. Afterward, the loaded SPI-GA-functionalized Car beads were washed to discard any un-linked βGL entities.

#### βGL activity estimation

The activities of both the free and the iβGLs were estimated. In case of the iβGL, 0.06 g of the βGL-loaded SPI-GA-functionalized Car beads were suspended in 0.5-ml citrate–phosphate buffer (pH 4.6; 0.1 M). As for the free βGL, it was dissolved in 0.5 ml of the aforementioned buffer. The utilized substrate solution was a 200-mM lactose solution which was prepared in citrate–phosphate buffer (pH 4.6; 0.1 M). The catalytic reaction was conducted via mixing the 0.5-ml buffer, which contained either the free or the iβGL, with 3.5 ml of the 200-mM lactose solution. The reaction proceeded for 15 min at 37 °C in a shaking water bath. Afterward, a portion of the reaction mixture was removed and was placed in a boiling water bath for around 10 min. After cooling to room temperature, the glucose content of the reaction mixture was evaluated while utilizing commercial glucose kits. One βGL unit (U) indicated that 1 μmol glucose/min was procured while performing the abovementioned catalytic reaction.

#### Thermal stability and thermodynamic parameters estimation for free and iβGL specimens

The free and the iβGL specimens were dissolved or suspended, respectively, in 0.1 M citrate–phosphate buffer (pH 4.6). These specimens were then subjected to thermal incubations at 56–65 °C temperature range. After predetermined durations, the βGL specimens were removed from the thermal incubations and were instantly assayed. The recorded activities were presented relative to the commencing activities which were assessed with no thermal incubation. Plots of log (residual activity percents) against time were then constructed, at each inspected temperature, and the first-order thermal denaturation rate constants (k_d_) were procured from the slopes. The procured k_d_ values were exploited to derive the half-lives (t_1/2_) and *D*-values of the βGL specimens as follows (Abdel-Wahab et al. 2018):

1 $$ t_{{{1}/{2}}} = {\text{ln 2}}/k_{d} , $$

2 $$ D{\text{-value}} = {\text{ ln 1}}0/k_{d} . $$

Plot of Log *D*-values against temperature (°C) was also constructed, and its slope amounted to − 1/*z* where *z* was the temperature increment that would induce a tenfold fall-off in the *D*-values. Furthermore, ln *k*_*d*_ values were plotted vs 1/temperature (1/K), and the attained slope corresponded to − *E*_*d*_/*R* where *E*_*d*_ was the activation energy of βGL thermal denaturation. The alterations in enthalpy (ΔH), entropy (ΔS), and Gibb’s free energy (ΔG) betwixt the active βGLs and their thermally denatured analogues were also calculated as follows:

3 $$ \Delta H = E_{d} {-} \, RT, $$

4 $$ \Delta G = \, - RT{\text{ ln }}\left( {k_{d} *h/k_{B} *T} \right), $$

5 $$ \Delta S = \, \left( {\Delta H{-} \, \Delta G} \right)/T, $$

*T* was the temperature in *K*, *R* was the universal gas constant (8.314 Jmol^−1^ K^−1^), *h* was the Planck constant (11.04 * 10^–36^ Jmin), and *k*_*B*_ was the Boltzmann constant (1.38*10^–23^ JK^−1^).

#### Storage stability of iβGL

A batch of the βGL-loaded SPI-GA-functionalized Car beads was prepared. Some of these beads were assayed directly after finishing the βGL loading process (100% activity), and the rest of the beads were placed in distilled water and kept in fridge. After specific periods, some of the stored beads were assayed, and their activity was presented relative to the 100% commencing activity.

#### Approaches adopted to boost the thermal stability of the iβGL

Four approaches were adopted to boost the thermal stability of the iβGL. Two of these approaches utilized the regular βGL-loaded SPI-GA-functionalized Car beads which were prepared and loaded as mentioned above.

- In the first approach, the βGL-loaded beads were soaked for 2 h within a 0.2 M glycine solution that was prepared in 0.1 M citrate–phosphate buffer (pH 4.6). The beads were then meticulously washed to remove excess glycine. Afterward, some of these beads were assayed (100%), and the remaining beads were thermally incubated at 56 °C. After predetermined durations, some of the βGL-loaded beads were removed from the thermal incubation and were instantly assayed. The recorded activities were then presented relative to the commencing 100% activity.
- In the second approach, the βGL-loaded beads were thermally incubated at 56–65 °C temperature range while being soaked in a 200-mM lactose solution. This lactose solution was prepared in 0.1 M citrate–phosphate buffer (pH 4.6). After predetermined durations, the iβGL specimens were removed from the thermal incubation, and the lactose was quickly and meticulously eliminated via washing before estimating the residual iβGL activities. Such residual activities were given relative to the activity of an iβGL specimen which was analogously soaked in lactose but was not thermally incubated.

The two other adopted approaches required modifying the SPI-GA-functionalized Car beads via altering the functionalizing GA concentration from the regular 25–5 or 10%. The 5 and 10% GA-functionalized beads were then loaded with βGL, and their thermal stabilities were estimated at 56 °C.

#### Storage stability of lactose treated iβGL (LT-iβGL)

The storage stability of the LT-iβGL was also investigated. Some of the βGL-loaded SPI-GA-functionalized Car beads were initially assayed (100% activity). The rest of the βGL-loaded beads were kept in fridge while being soaked in a 200-mM lactose solution, which was prepared in 0.1 M citrate–phosphate buffer. After specific periods, some of the lactose stored βGL-loaded beads were rigorously washed to eliminate any lactose residues. The beads were then assayed, and the recorded activities were presented relative to the 100% commencing activity.

#### GOS preparation and analysis

The βGL-loaded SPI-GA-functionalized Car beads were added to a 40% lactose solution so that a ratio of 1.30 U βGL: 1 g lactose was attained. A shaker incubator was then utilized to conduct the βGL transgalactosylation reaction at 50 °C and 100 rpm. After specific durations, samples were collected, and they were suitably diluted with water to spot them on TLC plates. Raffinose, a trisaccharide, was also spotted on the TLC plates. Propanol/water (85:15, *v*/*v*) solvent system was utilized to develop the plates. The locations of the GOS spots were visualized via spraying the plates with a 0.5% 1-naphthol solution, which was solvated in 5% ethanolic sulfuric acid solution, and subsequently heating them. Such locations were marked in analogous unsprayed TLC plats. The unsprayed plates were then put within the Advion plate express, and the GOS spots were extracted via methanol. Afterward, they were analyzed with an Advion compact mass spectrometer with m/z expression range 10–1200. The rate within the spectrometer was put at 0.2 ml/min, and moderate voltage and temperature were adopted (typical fragmentation mode). Finally, positive ion electrospray ionization (ESI +) and negative ion electrospray ionization (ESI −) mass charts were acquired.

## Results and discussion

### Thermal stability and thermodynamic parameters estimation for free and iβGL specimens

**Fig. 1:**
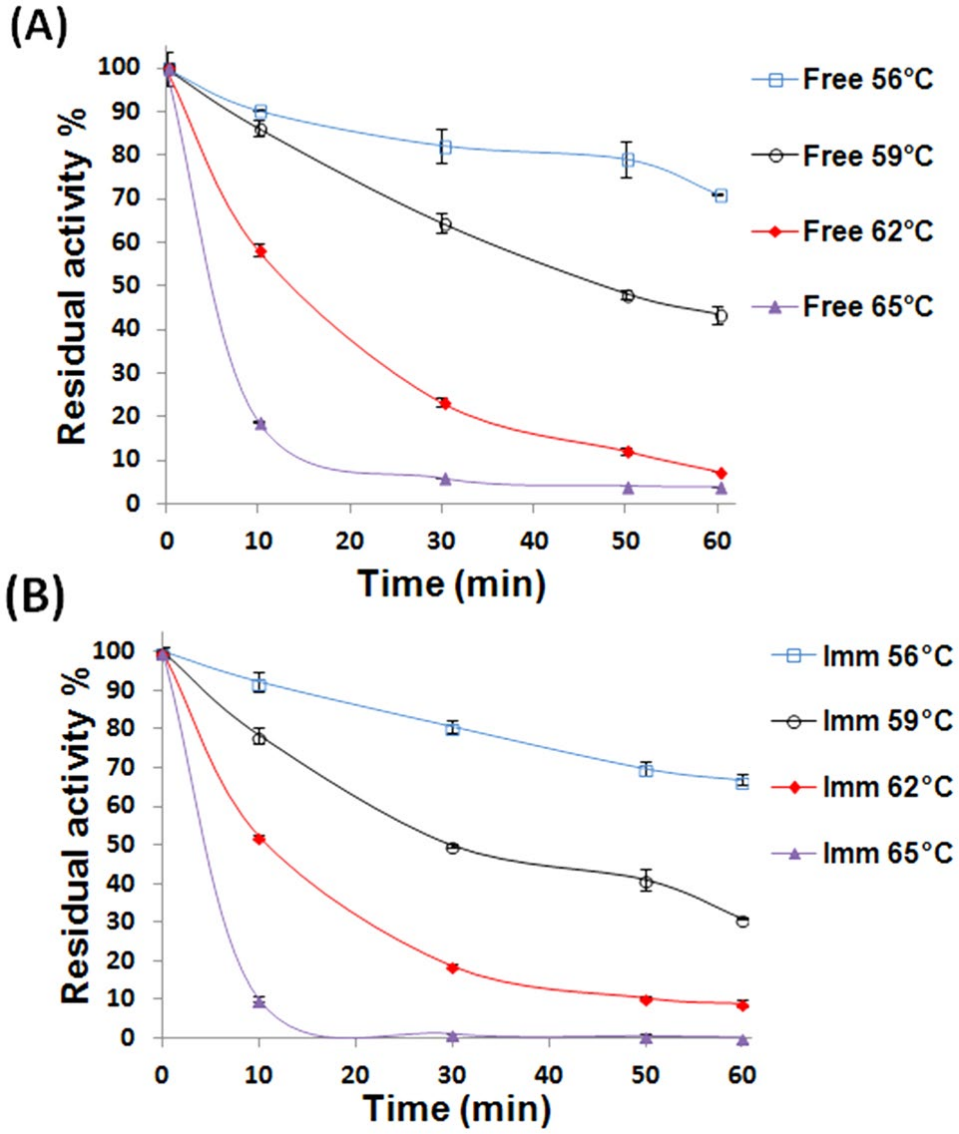
Thermal stability of free and iβGLs. Relative activity percents kept by the free βGL (**A**) and iβGL (**B**) following their thermal incubation at the given temperatures

**Fig. 2:**
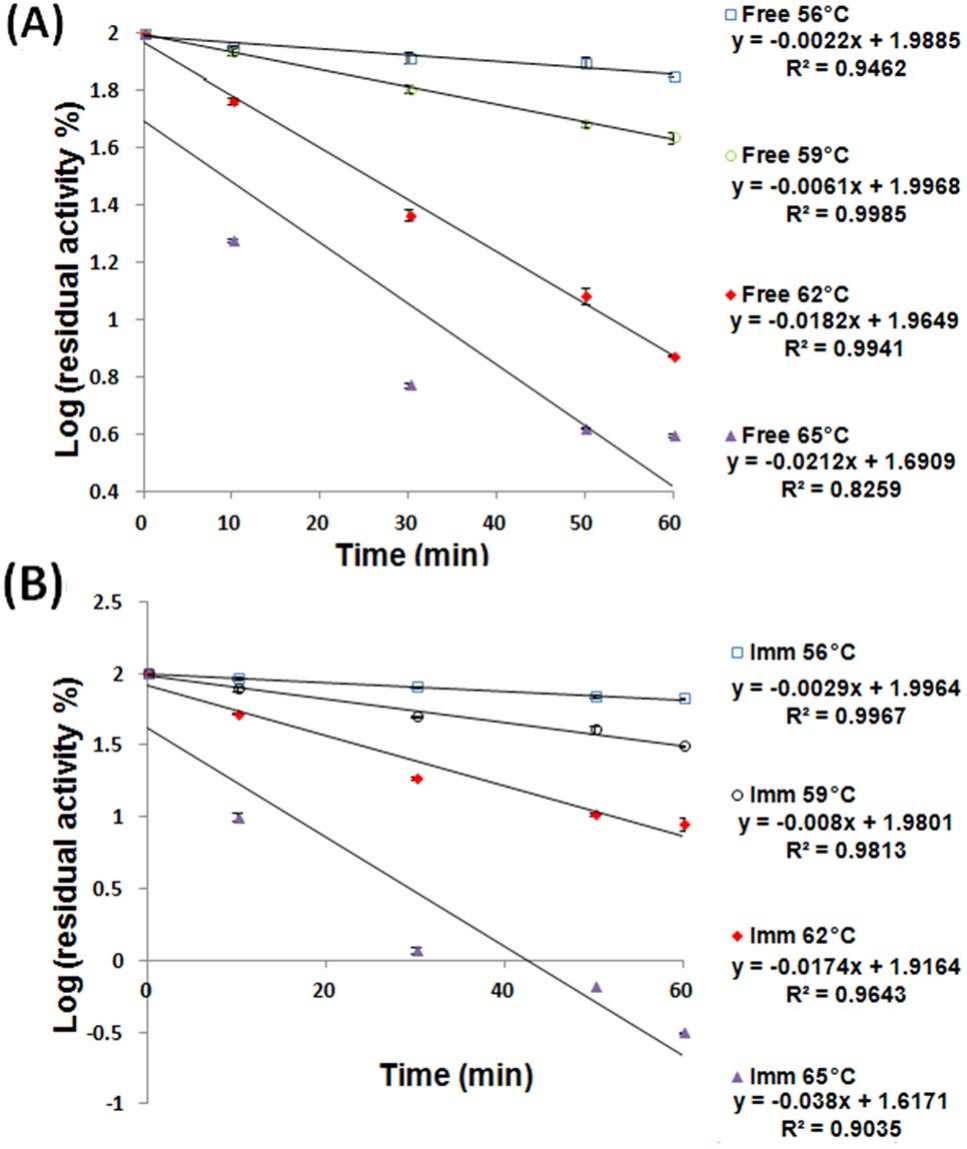
Estimation of *k*_*d*_ values for free and iβGLs. The Log (residual activity percents) presented by the free βGL (**A**) and the iβGL (**B**) were plotted against time to calculate the *k*_*d*_ values (-slope)

Figure 1A disclosed that after thermal incubations, the free βGL, mostly, retained higher activity percents than those retained by the iβGL (Fig. 1B). This might reflect the impaired thermal stability of the iβGL. Such impaired thermal stability could be regarded to the uncontrolled interactions that occurred amid the iβGL, and the immobilizer as the uncontrolled enzyme-immobilizer interactions was formerly debated to influence the enzyme stability (Barbosa et al. 2014; Bolivar et al. 2022; Jia et al. 2020). In order to further clarify the status of the iβGL thermal stability as compared to the thermal stability of its free analogue, the *k*_*d*_ values were estimated for both βGL specimens (Fig. 2). Afterward, the *k*_*d*_ values were exploited to estimate the t_1/2_ and *D*-values (Eqs. 1 & 2). AT 56, 59, and 65 °C, the iβGL presented loftier k_d_, and lesser t_1/2_ and *D*-values (Table 2) which indicated that it exhibited a more prominent thermal irreversible denaturation (Da Silva et al. 2018), and that shorter thermal incubations were required to trigger 50% and 90% declines in its presented activities, respectively (Marangoni 2003). As for the 62 °C temperature, the iβGL presented a slightly lower *k*_*d*_, and slightly loftier *t*_1/2_ and *D*-value which indicated that it possessed a slightly loftier thermal stability at 62 °C.

**Table 2:** Thermodynamic parameters calculated for the free βGL, the iβGL, and the LT- iβGL

	Free βGL	iβGL	LT-iβGL
56 °C			
*k*_*d*_ (min^−1^)	0.0022	0.0029	0.0019
* t*_1/2_ (min)	321.08	235.01	368.83
*D*-value (min)	1066.62	780.70	1225.23
Δ*H*° (kJ mol^−1^)	242.9	257.78	109.30
Δ*G*° (kJ mol^−1^)	108.88	108.03	109.26
Δ*S*° (J mol^−1^ K^−1^)	407.16	454.97	0.12
59 °C			
*k*_*d*_ (min^−1^)	0.0061	0.008	0.0028
* t*_1/2_ (min)	112.96	86.17	245.44
* D*-value (min)	375.24	286.26	815.34
Δ*H*° (kJ mol^−1^)	242.88	257.76	109.28
Δ*G*° (kJ mol^−1^)	107.02	106.27	109.16
Δ*S*° (J mol^−1^ K^−1^)	409.03	456.08	0.36
62 °C			
*k*_*d*_ (min^−1^)	0.0182	0.0174	0.0033
* t*_1/2_ (min)	38.03	39.74	212.84
*D*-value (min)	126.34	132.02	707.04
Δ*H*° (kJ mol^−1^)	242.85	257.73	109.25
Δ*G*° (kJ mol^−1^)	104.98	105.10	109.77
Δ*S*° (J mol^−1^ K^−1^)	411.38	455.42	− 1.55
65 °C			
*k*_*d*_ (min^−1^)	0.0212	0.038	0.006
*t*_1/2_ (min)	32.63	18.24	115.10
* D*-value (min)	108.4	60.59	382.37
Δ*H*° (kJ mol^−1^)	242.83	257.71	109.23
Δ*G*° (kJ mol^−1^)	105.51	103.87	109.05
Δ*S*° (J mol^−1^ K^−1^)	406.08	454.93	0.52

**Fig. 3:**
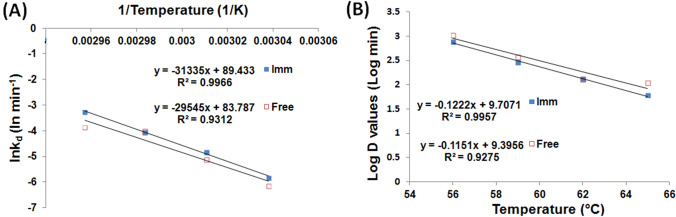
Estimation of *E*_*d*_ and *z*-values for free and iβGLs. **A** Arrhenius plot exploited to calculate the *E*_*d*_ for the free and iβGL specimens (**B**) log (*D*-values) plotted against temperature to calculate the *z*-values for the free and iβGL specimens

Arrhenius plot (Fig. 3A) was then constructed to derive the *E*_*d*_ values which amounted to 245.64 and 260.52 kJ mol^−1^ for the free and iβGLs, respectively. The heightened *E*_*d*_ presented by the iβGL indicated that a larger quantity of energy would be necessary to cause its denaturation (Wahba 2020). Noteworthy, heightened *E*_*d*_ values were frequently reported following immobilization, and this was considered as an indication of the immobilized enzymes boosted thermal stability (Agrawal et al. 2020; Sadaqat et al. 2022; Wahba 2020). Afterward, the enthalpy of denaturation (ΔH), which reflected the entire sum of energy needed to denature an enzyme (Da Silva et al. 2018) at any specified temperature, was estimated. However, it should be noted that Δ*H* was directly derived from the *E*_*d*_ (Eq. 3). Thus, as was the case with the *E*_*d*_ values, the iβGL presented loftier Δ*H* values than its free analogue. The entropy of denaturation (Δ*S*) was also listed in Table 2. Δ*S* values give an indication of the randomness within a system, and they are usually incremented upon denaturation. Hence, the loftier the Δ*S* values, the lower the stability of the enzyme (Marangoni 2003). The iβGL presented loftier Δ*S* values which marked its lower stability.

The *z*-values are also estimated from Fig. 3B, and they amounted to 8.69 and 8.18 °C in case of the free and iβGLs, respectively. Thus, a slightly lower temperature heightening was needed to induce 90% fall-off in the iβGL *D*-values, and this indicated that it was a little more sensitive to temperature heightening that its free analogue. Noteworthy, the immobilized pectinase and the treated gellan gum iβGL also offered lower *z*-values as compared to their free analogues (Abdel-Wahab et al. 2018; Wahba 2020).

From the data presented so far, it could be seen that the status of the iβGL thermal stability was questionable. Most of the *k*_*d*_, *t*_1/2_ and *D*-values indicated the iβGL thermal stability was lower than that of its free analogue. Such lower thermal stability was further confirmed from the iβGL loftier Δ*S* values. On the other hand, the *E*_*d*_ and Δ*H* values reflected the increased thermal stability of the iβGL. Thus, the Gibb’s free energy (Δ*G*) was considered. Δ*G* is a more dependable enzyme stability indicator as it comprises the enthalpic and the entropic contributions together (Marangoni 2003). The loftier Δ*G* implies that the enzyme is more resistant to denaturation and is more thermo-stable (Da Silva et al. 2018). The free βGL presented loftier ΔG at all temperatures except at 62 °C. At 62 °C, the iβGL presented a Δ*G* which was 0.12 kJ mol^−1^ loftier than that presented by its free analogue (Table 2). Noteworthy, when the immobilization was proven to boost the thermal stability of *β*-amylase, lipase, carboxymthyl-cellulase, mannanase, βGL, and protease, the immobilized preparations exhibited loftier ΔG at all listed temperatures (Agrawal et al. 2020; Ferreira et al. 2018; Karim et al. 2021; Sadaqat et al. 2022; Wahba 2020, 2022b). Thus, it could be concluded that the iβGL presented a lower thermal stability than did its free analogue. This lower thermal stability could be regarded to the uncontrolled interactions which occurred amid the iβGL and the SPI-GA-functionalized Car beads. Noteworthy, the thermal stability of the immobilized *Bacillus circulans* βGL was improved after blocking the immobilizer (Eupergit C) remaining epoxy moieties via incubation in glycine (Torres and Batista-Viera 2012). Blocking the immobilizer remaining covalently reactive moieties probably hampered the occurrence of the uncontrolled enzyme-immobilizer interactions, and this eventually boosted the thermal stability of the Eupergit C iβGL. Thus, the uncontrolled interactions amid the SPI-GA-functionalized Car beads and the iβGL should be hampered to boost the iβGL thermal stability.

### Storage stability of iβGL

**Fig. 4:**
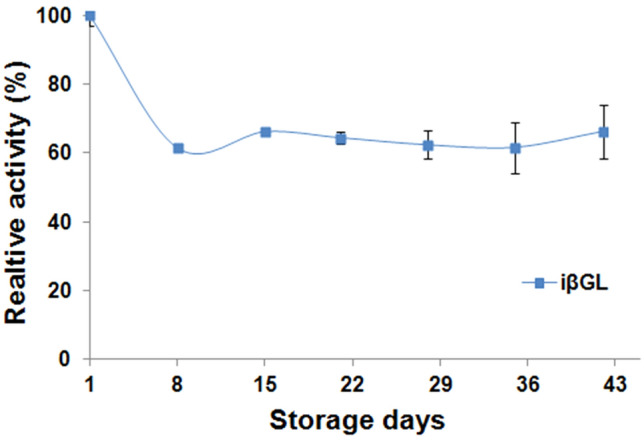
Storage stability of iβGL. Relative activity percents kept by the iβGL after its storage in the fridge

The iβGL suffered a sharp fall-off in its activity on its 8th storage day, and only 61.39% of its commencing activity was presented (Fig. 4). Such a sharp initial fall-off in the iβGL activity could be referred to the uncontrolled interactions which occurred amid the iβGL and the SPI-GA-functionalized Car beads. These uncontrolled interactions were formerly debated to influence the enzyme stability (Barbosa et al. 2014; Bolivar et al. 2022; Jia et al. 2020). It should also be noted that the enzyme configuration could be altered secondary to its interactions with the carrier (Rodrigues et al. 2013). If these configurational alterations influenced the active site, then the enzyme would be deactivated, and this was probably the cause of the deactivation of a substantial iβGL amount during storage. Noteworthy, it was formerly debated that some of the βGL moieties, which were immobilized via the GA-functionalized chitosan beads, got deactivated upon extending the enzyme loading step owing to the uncontrolled enzyme-immobilizer interactions (Wahba 2018).

Upon further prolonging the iβGL storage, its activity fluctuated slightly, and 66.24% of the commencing activity was presented on the 42nd storage day (Fig. 4). This activity percent was close to the 65.69% activity presented on the 45th storage day of the *Bifidobacterium bifidum* βGL which was covalently conjugated to the processed SiO_2_ nanoparticles (Tizchang et al. 2020). Moreover, it was also comparable to the activity presented after storing the *Aspergillus oryzae* βGL, which was covalently conjugated to tannic acid stabilized silver nanoparticles, for 40 days (Arsalan et al. 2020).

Based on the above presented data, it could be concluded that the thermal and storage stabilities of the SPI-GA-functionalized Car beads iβGL were somewhat impaired. Hence, approaches should be adopted to boost the iβGL stability. Noteworthy, various approaches were formerly adopted to boost the stability of βGL (Table 3). These approaches included the incorporation of various additives, such as lactose (Warmerdam et al. 2013), galactose, raffinose, (Wahba 2015), various oligosaccharides (Deng et al. 2019), and also Mg^2+^ (Zhou et al. 2017). Immobilization was also shown to be a proficient βGL stabilization tool (Bayramoglu et al. 2023; Todea et al. 2021; Tizchang et al. 2020; Wahba 2020, 2021). Nonetheless, all the aforementioned stabilization approaches aimed at overcoming the inherent instability of βGL as enzymes are known to be somewhat unstable (Rodrigues et al. 2021). In the case in hand, the impaired thermal and storage stabilities of the SPI-GA-functionalized Car beads iβGL were regarded to the utilized immobilizer. The active functionalities in the SPI-GA-functionalized Car beads induced uncontrolled enzyme-immobilizer interactions, and these interactions destabilized the iβGL. Thus, the stabilizing approaches adopted herein aimed at inhibiting the occurrence of such uncontrolled interactions.

**Table 3:** Thermodynamic parameters presented by the iβGL at 56 °C after being subjected to various approaches to boost its stability

Approach to boost thermal stability of the iβGL	Thermodynamic parameters attained at 56 °C		
	*k*_*d*_ (min^−1^)	*t*_1/2_ (min)	*D* value (min)
Treating the loaded beads with glycine	0.0050	138.87	461.32
Altering the GA concentration to 5%	0.0073	94.71	314.63
Altering the GA concentration to 10%	0.0078	88.31	293.35
Treating the loaded beads with lactose	0.0019	368.83	1225.23

### Approaches adopted to boost the stability of the iβGL

Variable approaches were adopted to boost the stability of iβGL and prohibit the occurrence of post-immobilization uncontrolled interactions amid the iβGL and the SPI-GA-functionalized Car beads. Uncontrolled enzyme-immobilizer interactions could be prohibited if, after completing the immobilization process, the immobilizer was inert with no capability to establish any new physical or covalent linkages with the immobilized enzyme (Barbosa et al. 2014; Rodrigues et al. 2021). In case of the SPI-GA-functionalized Car beads, both SPI and GA could establish new linkages with the iβGL after finishing the immobilization process. SPI is a protein, and its iso-electric point was reported to be 4.5–4.6 (Tavernier et al. 2017; Wee et al. 2017). Such pH values were close to the 4.6 pH value adopted during the various reactions of the iβGL. Thus, SPI would present cationic and anionic entities that might establish new ionic linkages with the iβGL, and these newly formed ionic linkages could destabilize the iβGL. As regards to GA, it could establish new covalent linkages with the iβGL. Nonetheless, it should be noted that the physical reactivity of SPI could not be neutralized. It was formerly mentioned that an immobilizer with physically active surface functionalities would always exhibit a physically active surface (Virgen-Ortíz et al. 2017). On the other hand, any remaining covalently reactive GA entities could be blocked and neutralized via the interaction with glycine (Wahba and Hassan 2017).

**Fig. 5:**
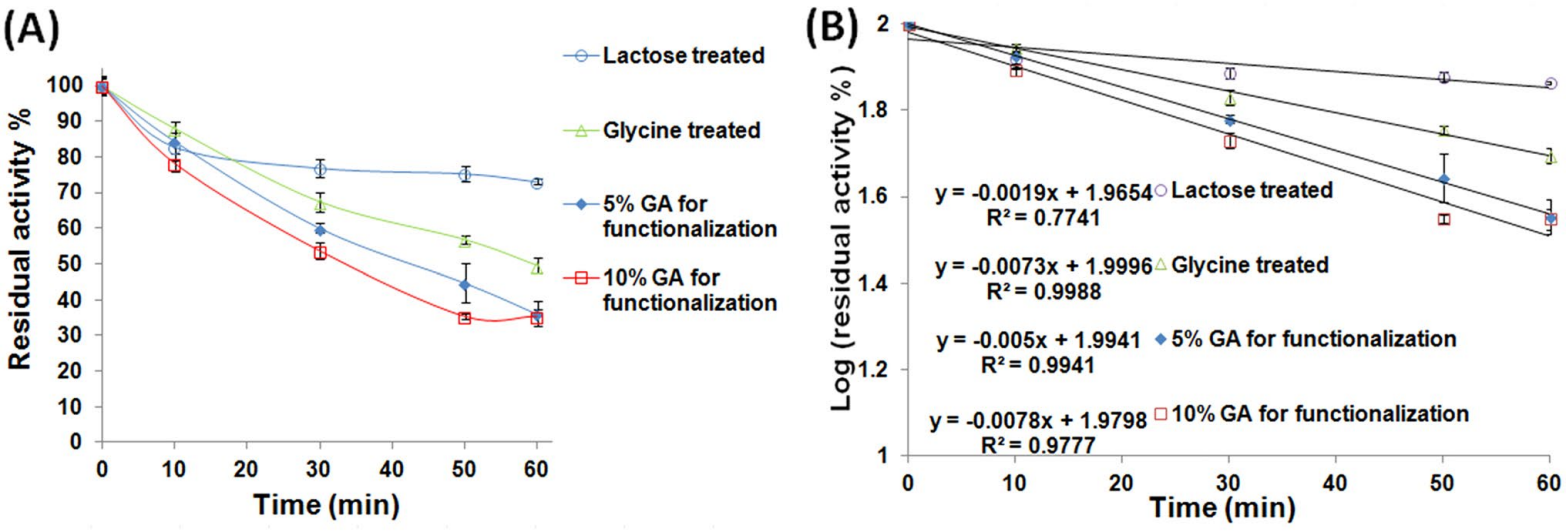
Thermal stability and estimation of *k*_*d*_ values for the variously processed iβGL specimens. **A** The thermal stability of the iβGL specimens, which were variously processed to boost their stability, was represented by the residual activity percents presented by the respective iβGL specimens after 56 °C thermal incubation. **B** The Log values of these (residual activity percents) were then plotted against time to calculate the *k*_*d*_ values (-slope)

Thus, we first attempted to render the SPI-GA-functionalized Car beads covalently inert via the interaction with glycine. It was previously shown that a 0.4:1 molar ratio of glycine to GA could neutralize the hazardous effect of GA in water within just 30 min (Chen and Roberts 2002). Noteworthy, a 25% GA (2.5 M) was adopted while preparing the SPI-GA-functionalized Car beads. Nonetheless, the quantity of GA attached to the SPI-GA-functionalized Car beads was analogous to that attached to the agar-Car disks which were functionalized with only a 3% GA (0.3 M) solution (Wahba 2022a). Moreover, a substantial fraction of the attached GA entities would already be covalently linked to the iβGL. Accordingly, not much GA entities would be free and covalently reactive after finishing the βGL immobilization process. Hence, 0.2-M glycine would provide enough entities to block and neutralize the unreacted GA within the loaded SPI-GA-functionalized Car beads. However, after treating the loaded SPI-GA-functionalized Car beads with 0.2-M glycine for 2 h, no improvement was recorded in the thermal stability of the iβGL. The glycine-treated iβGL still retained lower activity percents (Fig. 5A) than those retained via the free βGL after the 56 °C thermal incubation (Fig. 1A). The k_d_ was then estimated for the glycine-treated iβGL (Fig. 5B), and it was loftier than that recorded for the free βGL. Moreover, the glycine-treated iβGL *t*_1/2_ and *D*-values were lesser (Table 4) than those presented by the free βGL (Table 2). Hence, it could be concluded that the thermal stability of the glycine-treated iβGL was still impaired. On the other hand, blocking the remaining vinyl-sulfone moieties of the divinyl-sulfone-functionalized agarose via incubation in glycine boosted the thermal stability of immobilized lipase (Dos Santos et al. 2015) and the stability of immobilized penicillin G acylase (Da Rocha et al. 2022). Moreover, utilizing glycine to block the immobilizer remaining epoxy moieties boosted the thermal stabilities of the ES-105 immobilized transaminase (Jia et al. 2020) and the Eupergit C iβGL (Torres and Batista-Viera 2012) (Table 1). Accordingly, the inability of glycine to improve the impaired thermal stability of the SPI-GA-functionalized Car iβGL could indicate that the uncontrolled enzyme-immobilizer interactions suffered by this iβGL were not mediated via GA.

**Table 4:** Various approaches which were formerly adopted to boost the stability of βGL

Microbial βGL source	Method of stabilization	Improvement in βGL stability	Reference
*A. oryzae*	Immobilization via GA-functionalized diaminopolyethylene glycol-polydopamine-coated magnetic nanoparticles	• Compared to the free βGL, the iβGL was more thermo-stable at 55 and 65 °C	Bayramoglu et al. (2023)
*A. oryzae*	Immobilization via amino-activated Ni-Zn magnetic particles	• Compared to the free βGL, the iβGL was more thermo-stable at 50 and 60 °C	Todea et al. (2021)
*Bifidobacterium bifidum*	Immobilization via GA-processed SiO_2_ nanoparticles	• The iβGL kept 78.23% of its activity after 10-h thermal incubation at 50 °C • The iβGL kept 65.69% of its activity after 45 days of storage	Tizchang et al. (2020)
*A. oryzae*	Immobilization via GA-egg white protein processed gellan gum beads	• Compared to the free βGL, the iβGL offered smaller k_d_ and bigger *t*_1/2_, *D*-values, Δ*H*, and Δ*G* values • The iβGL kept 87.64% of its activity after 63 days of storage	Wahba (2020)
*A. oryzae*	Immobilization via GA-polyethyleneimine-processed carrageenan-calcium pectinate beads	• Compared to the free βGL, the iβGL offered smaller *k*_*d*_ and bigger *t*_1/2_, *D*-values, ΔH, and ΔG values • The iβGL kept 90.43% of its activity after 43 days of storage	Wahba (2021)
*Escherichia coli*	The addition of various oligosaccharides (OS), such as isomalto-OS, Xylo-OS, Konjac-OS, or Mycose	• The treated βGL specimens retained higher activity after their thermal incubations at 60 °C	Deng et al. (2019)
*A. oryzae*	The addition of galactose or raffinose	• The βGL samples retained higher activity after their thermal incubations at 60 °C	Wahba (2015)
*Bacillus circulans*	The addition of 5 or 30% lactose	• The treated βGL offered higher *t*_1/2_ values at 40 and 60 °C	Warmerdam et al. (2013)
*Bacillus megaterium*	The incorporation of 5 mM Mg^2+^	• The treated βGL exhibited higher stability in aqueous hydrophilic solvents as shown by its extended *t*_1/2_ values	Zhou et al. (2017)

Another approach was then attempted which also involved the GA entities. This approach involved altering the concentration of the functionalizing GA solution. GA concentration is amid the factors that determine the degree of polymerization of the amino bound GA entities where raising the GA concentration would increment its polymerization extent (Barbosa et al. 2014). Thus, reducing the GA concentration to 10 and 5% might alter the GA configuration, and this might eventually alter the GA-βGL interactions. Nonetheless, no improvement was recorded in the thermal stability of the βGL samples immobilized via the 5 and 10% GA processed SPI-GA-functionalized Car beads. They still offered (Fig. 5A) lower activity percents than those offered via the free βGL following the 56 °C thermal incubation (Fig. 1A). Moreover, their k_d_ values were higher and their *t*_1/2_ and *D*-values were lower (Table 4) than those offered by the free βGL (Table 2).

Since no improvements were attained in the stability of the iβGL after implementing the two aforementioned GA-related approaches, it could be concluded that GA post-immobilization covalent interactions were not responsible for the reduced stability of the iβGL. Such reduced stability would probably be a consequence of the physical interactions established betwixt the iβGL and the SPI coating, which would present both anionic and cationic entities (iso-electric point of 4.5–4.6 (Tavernier et al. 2017; Wee et al.^2017^). Nonetheless, as mentioned earlier, such physical reactivity could not be neutralized. Thus, to stabilize the iβGL and keep its activity, stabilizers were added to the iβGL sample. Substrates are regarded as enzymes stabilizers. The specific and robust binding amid the substrates and the native conformation of their enzyme reduces the possibility of thermal denaturation presumably owing to conformational tightening (Lejeune et al. 2001). Furthermore, the βGL substrate, lactose, was formerly reported to boost the thermal stability of altered βGLs (Illeová and Polakovič 2018; Warmerdam et al. 2013). Thus, the 56 °C thermal incubation of the iβGL was accomplished while placing the iβGL in a lactose solution. Afterward, the lactose was discarded via meticulous washing, and the residual iβGL activity was assessed. Lactose managed to boost the iβGL thermal stability. The lactose-treated iβGL (LT-iβGL) kept 73.15 ± 0.63% activity (Fig. 5A), whereas 71.02 ± 0.17% activity (Fig. 1A) was kept by the free βGL following 1 h incubation at 56 °C. Moreover, the LT-iβGL presented a lower *k*_*d*_ value and larger *t*_1/2_ and *D*-values (Table 4) than those offered by the free βGL (Table 2). Thus, the thermal stability of the LT-iβGL was further investigated to verify its boosted thermal stability.

### Assessment of LT-iβGL thermal stability at various temperatures and estimating the thermodynamic parameters

**Fig. 6:**
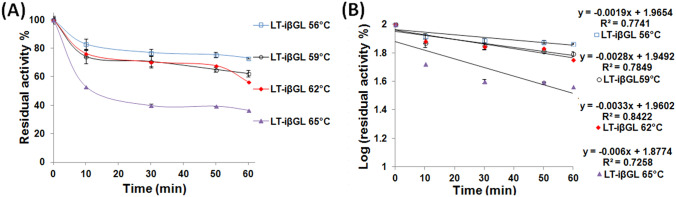
Thermal stability and estimation of *k*_*d*_ values for the LT-iβGL. **A** Thermal stability offered via the LT-iβGL and presented as the activity percents kept following the incubation at the given temperatures. **B** The Log (residual activity percents) of the LT-iβGL which were plotted against time to calculate the *k*_*d*_ values (-slope)

Figure 6A unveiled the superior thermal stability of the LT-iβGL. For instance, 62.20 ± 2.26% activity was kept by the LT-iβGL, whereas only 43.43 ± 2.05% activity was kept by the free βGL following 1 h incubation at 59 °C (Fig. 1A). This LT-iβGL thermal stability was comparable to that presented by the amino-activated Ni-Zn magnetic particles iβGL following 1 h incubation at 60 °C (Todea et al. 2021). Moreover, the LT-iβGL thermal stability was superior to that of the *Escherichia coli* βGL which was treated with various oligosaccharides (OS), such as isomalto-OS, Xylo-OS, Konjac-OS, or Mycose at a βGL: OS weight ratio of 1:500. The loftiest stability was recorded for the Konjac-OS treated βGL, which kept around 45% of its activity following 1 h incubation at 60 °C (Deng et al. 2019). On the other hand, 56.43% activity was kept by the LT-iβGL following 1 h incubation at 62 °C (Fig. 6A). The boosted thermal stability of the LT-iβGL was further verified from its *k*_*d*_ values which were estimated from Fig. 6B. The LT-iβGL offered smaller *k*_*d*_ values than those offered by the free βGL. The LT-iβGL also offered loftier t_1/2_ and *D*-values (Table 2). Similarly, the thermal stability of the *Bacillus circulans* βGL was boosted, and its 40 and 60 °C *t*_1/2_ values were heightened in the presence of lactose. This boosted thermal stability was regarded to the conjugation amidst the enzyme and its substrate (Warmerdam et al. 2013). It is worth mentioning that the 212.84 min *t*_1/2_ and the 707.04 min *D*-value recorded herein at 62 °C for the LT-iβGL were loftier than the 64.62 min *t*_1/2_ and the 214.65 min *D*-value recorded at 62 °C for the βGL which was immobilized via the egg white protein processed gellan gum beads (Wahba 2020). Moreover, the LT-iβGL 62 °C *t*_1/2_ and *D*-value were also loftier than the 103.36 min *t*_1/2_ and the 343.34 min *D*-value which were recorded at 62 °C for the Car-calcium pectinate beads iβGL (Wahba 2021). Thus, the LT-iβGL was more stable at such heightened temperature.

The sturdy binding of lactose to the iβGL native configuration probably prevented the destabilizing uncontrolled enzyme-immobilizer interactions, and this boosted the thermal stability of the LT-iβGL. Noteworthy, preventing the uncontrolled enzyme-immobilizer interactions was formerly accompanied with an improvement in the thermal stability of immobilized enzyme (Table 1). For instance, blocking the epoxy moieties of ES-105 with glycine boosted the thermal stability of the immobilized transaminase at 57 °C (Jia et al. 2020). Moreover, blocking the remaining epoxy moieties of epoxy grafted Purolite^®^A109 via incubation in phenylalanine boosted the thermal stability of immobilized Lipase. The blocked immobilized lipase kept 5.42-fold higher activity than that kept by its unblocked analogue after the incubation at 65 °C for 8 h, and this proved the boosted thermal stability of the blocked immobilized lipase (Mihailović et al. 2014). It should be noted that the stabilizing effect imparted herein via the incubation in lactose was more pronounced. The LT-iβGL kept 36.62% activity after 1 h incubation at 65 °C (Fig. 6A), whereas the untreated iβGL kept only 0.31% activity after akin incubation (Fig. 1B). On another occasion, blocking the remaining vinyl-sulfone moieties of the divinyl-sulfone-functionalized agarose via incubation in variable nucleophiles boosted the 60 °C thermal stability of the immobilized lipase. The *t*_1/2_ values of the glycine, ethylenediamine, and cysteine blocked immobilized lipases were 1.75-, 2.67-, and 5.25-fold higher than the t_1/2_ presented by the unblocked immobilized lipase (Dos Santos et al. 2015). In the case in hand, the LT-iβGL *t*_1/2_ values were 5.36- and 6.31-fold higher than the *t*_1/2_ values presented by the untreated iβGL at 62 and 65 °C, respectively (Table 2).

**Fig. 7:**
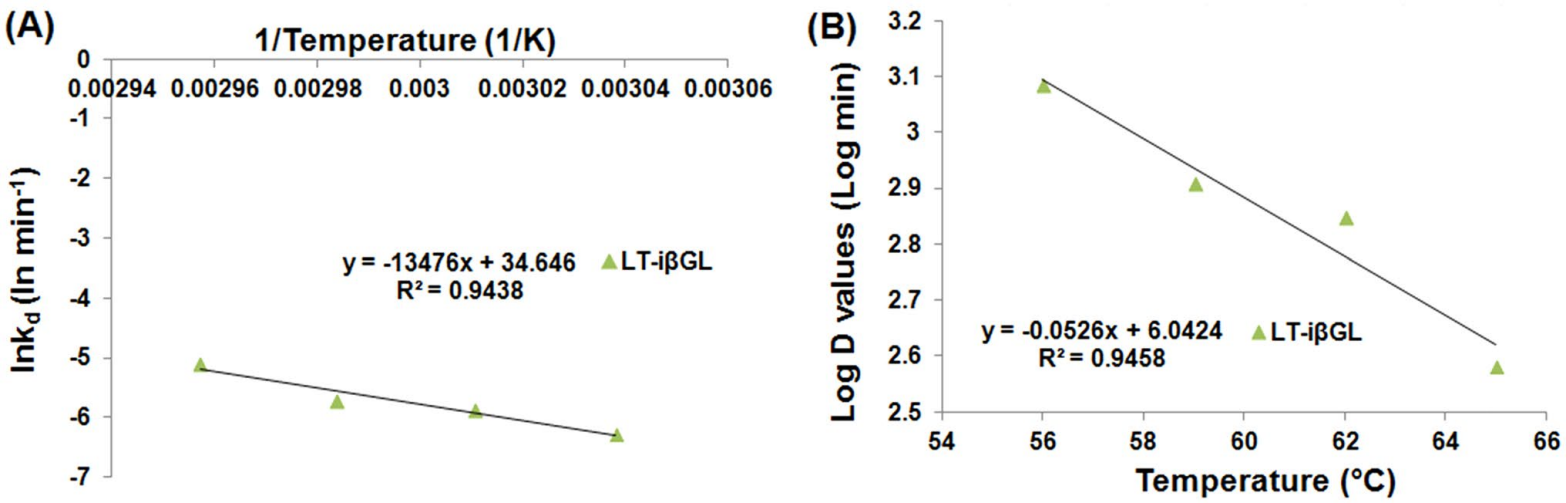
Estimation of *E*_*d*_ and *z*-values for the LT-iβGL. **A** Arrhenius plot exploited to calculate the *E*_*d*_ for the LT-iβGL. **B** log (*D*-values) plotted against temperature so as to calculate the *z*-values for LT-iβGL specimens

As regards to the *E*_*d*_ of the LT-iβGL, it was derived from Fig. 7A and it amounted to 112.04. This was much lower than the 245.64 kJ mol^−1^*E*_*d*_ recorded for the free βGL. Nevertheless, the decline in *E*_*d*_ in the presence of lactose was also noticed while investigating the thermal stability of *B. circulans* βGL. The thermal stability of *B. circulans* βGL was boosted via lactose presence. Nonetheless, its *E*_*d*_ dropped from 200 to 72 and 14 kJ mol^−1^ in the presence of 5% and 30% lactose, respectively. These *E*_*d*_ declines were accompanied with a decline in the temperature dependence of *B. circulans* βGL thermal inactivation (Warmerdam et al. 2013). *E*_*d*_ is estimated from the slope of the plot of ln(kd) vs 1/temperature. Thus, if the enzyme thermal inactivation became less dependent on temperature, it would be expected that the slope of the plot would attain a lesser absolute value, and accordingly, the *E*_*d*_ (slope =  – *E*_*d*_/*R*) would attain a lesser value. This debate could also be applied herein. The LT-iβGL thermal inactivation was less dependent on temperature, and this caused the slope in Fig. 7A to be less steep. Thus, a lesser *E*_*d*_ was acquired. Analogously, the protease immobilized via an activated gum tragacanth-agar carrier and the carboxymethyl-cellulase immobilized inside agarose presented *E*_*d*_ values which were significantly lower than those presented by their free analogues, and this was also regarded to the fact that the immobilized enzymes thermal inactivation became less temperature dependent (Karim et al. 2021; Wahba 2022b).

Δ*G* values were then exploited to confirm the boosted thermal stability of the LT-iβGL as Δ*G* is a more dependable enzyme stability indicator that comprises the enthalpic and the entropic contributions together (Marangoni 2003). The LT-iβGL presented loftier ΔG than did the free βGL, at all temperatures (Table 2), and this confirmed its boosted thermal stability and its heightened resistance to denaturation (Da Silva et al. 2018). Moreover, the LT-iβGL offered much lesser ΔS values than did the free βGL, and this indicated that the LT-iβGL was a more stable enzyme (Marangoni 2003). Similarly, the lesser ΔS values presented via the immobilized carboxymethyl-cellulase (Karim et al. 2021) and the immobilized protease (Wahba 2022b) indicated their boosted thermal stability. Thus, it could be concluded that the lactose treatment boosted the thermal stability of the iβGL.

The *z*-value was also estimated for the LT-iβGL (Fig. 7B), and it amounted to 19.01 °C (Table 2). This indicated that a whole 19.01 °C temperature heightening was needed to induce 90% fall-off in the LT-iβGL *D*-value, whereas only an 8.69 °C temperature heightening would be needed to induce such fall-off for the free βGL (*z*-value 8.69). This further verified that the thermal inactivation of the LT-iβGL was less affected and less dependent on temperature. Analogously, immobilizing protease via the activated gum tragacanth-agar raised its *z*-value from 8.70 to 27.62 °C owing to the lesser dependence of its thermal inactivation on temperature (Wahba 2022b). Moreover, immobilizing carboxymethyl-cellulase into agarose also raised its *z*-value from 22.78 to 36.82 °C (Karim et al. 2021).

### Storage stability of LT-iβGL

**Fig. 8:**
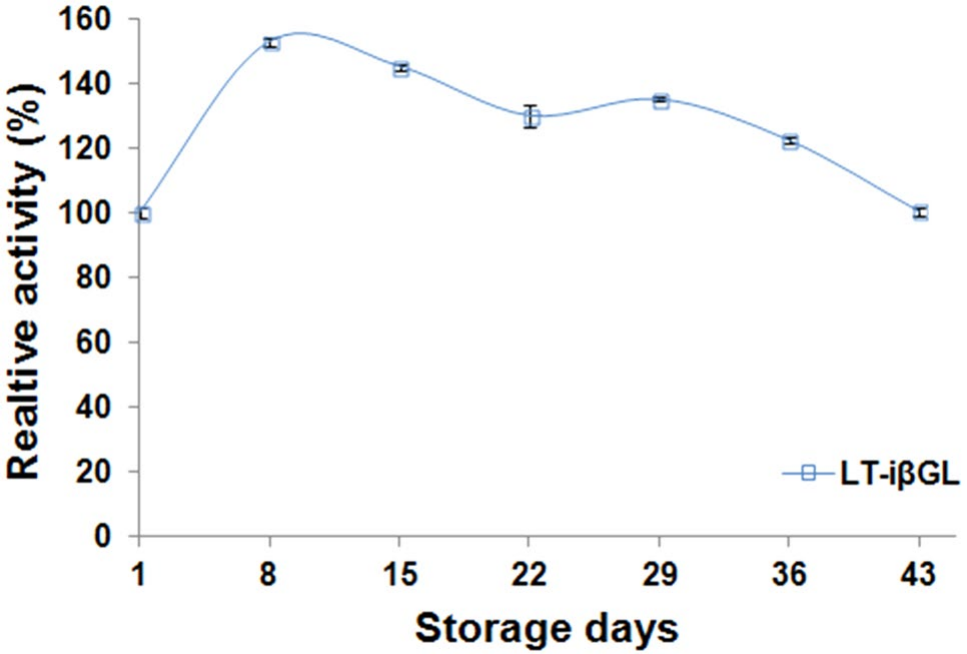
Storage stability of LT-iβGL. Relative activity percents kept by the LT-iβGL after its storage in the fridge

After storing the iβGL with lactose, its relative activity was significantly heightened and reached 152.80 ± 1.17% on the 8th storage day (Fig. 8). The heightening in the iβGL relative activity after its prolonged incubation with its substrate was formerly noticed while reusing the βGLs immobilized via the processed agar and the processed agar-Car beads. Such heightening was regarded to the enzyme attaining its uppermost efficiency after the incubation with its substrate (Wahba and Hassan 2017). It was also disclosed that upon extending the storage of the LT-iβGL, its relative activity gradually fell-off. However, a 100.41 ± 1.08% relative activity was presented on the 43^rd^ storage day, and this represented heightened storage stability. Thus, it could be implied that the robust binding amid lactose and the iβGL impeded the deactivating enzyme-immobilizer interactions and preserved the activity of the iβGL. On other occasions, 90.43% and ~ 91% relative activities were presented via the *Aspergillus oryzae* βGLs immobilized onto the Car-calcium pectinate beads, and the egg white protein processed gellan gum beads after 43 and 49 storage days, respectively (Wahba 2020, 2021). Moreover, only 77.2% activity was presented after storing the *Aspergillus oryzae* βGL, which was covalently conjugated to the GA-functionalized diaminopolyethylene glycol-polydopamine-coated magnetic nanoparticles, for 42 days (Bayramoglu et al. 2023).

### GOS formation

The GOS formed by the *A. oryzae* βGL were formerly shown to exhibit altered degrees of polymerization (DPN), such as GOS-3, GOS-4, GOS-5, and GOS-6 (Sass and Jördening 2020; Todea et al. 2021; Wahba 2021; Yin et al. 2017). The molecular formulas of these GOS are C_18_H_32_O_16,_ C_24_H_42_O_21,_ C_30_H_52_O_26,_ and C_36_H_62_O_31,_ respectively. Hence, they are expected to be presented at 504, 666, 828, and 990 m/z ratios, respectively. Nevertheless, they might appear at somewhat different *m*/*z* ratios as such GOS might get linked to the available anions or cations. Moreover, GOS might get de-protonated while undergoing the analysis (Juvonen et al. 2019; Neri et al. 2011). It should also be noted that more than one *m*/*z* peak could represent the same GOS DPN. For example, *m*/*z* peaks of 527.1 and 534.2 formerly indicated the presence of GOS-3 (Yin et al. 2017).

**Fig. 9:**
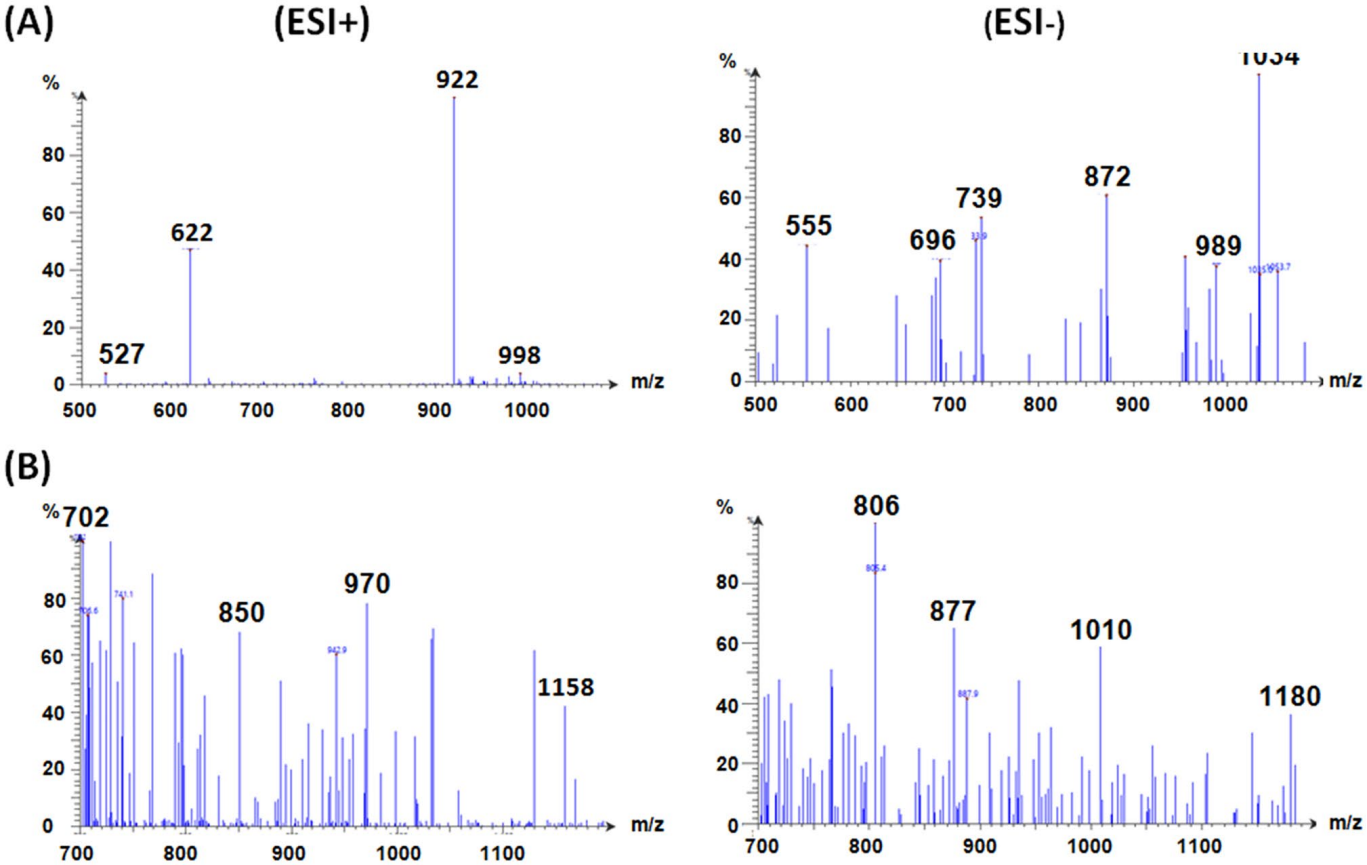
ESI-mass charts. Positive and negative ions ESI-mass charts recorded for GOS procured after 3 h (**A**) and 6 h (**B**)

**Fig. 10:**
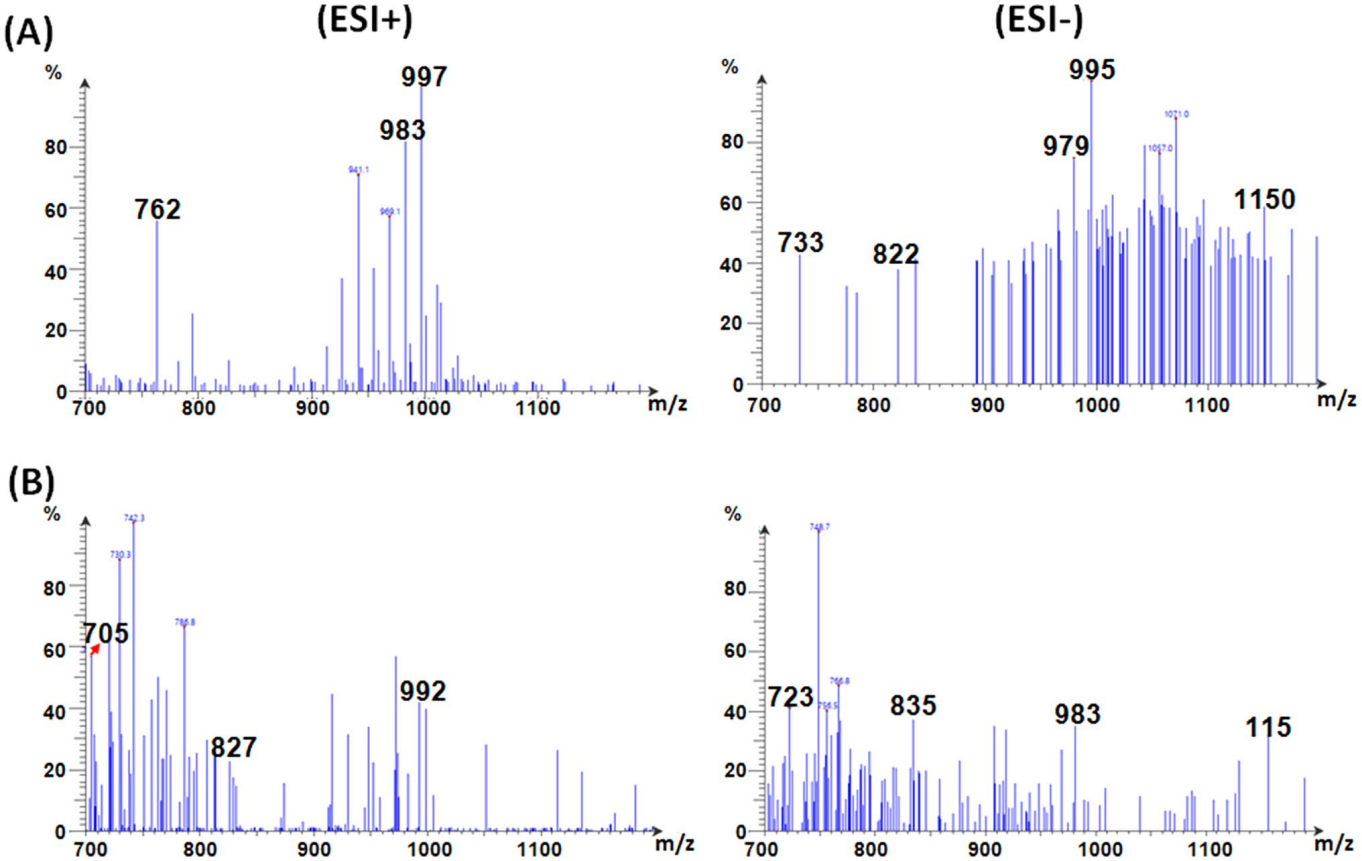
ESI-mass charts. Positive and negative ions ESI-mass charts recorded for GOS procured after 8 h (**A**) and 15 h (**B**)

**Fig. 11:**
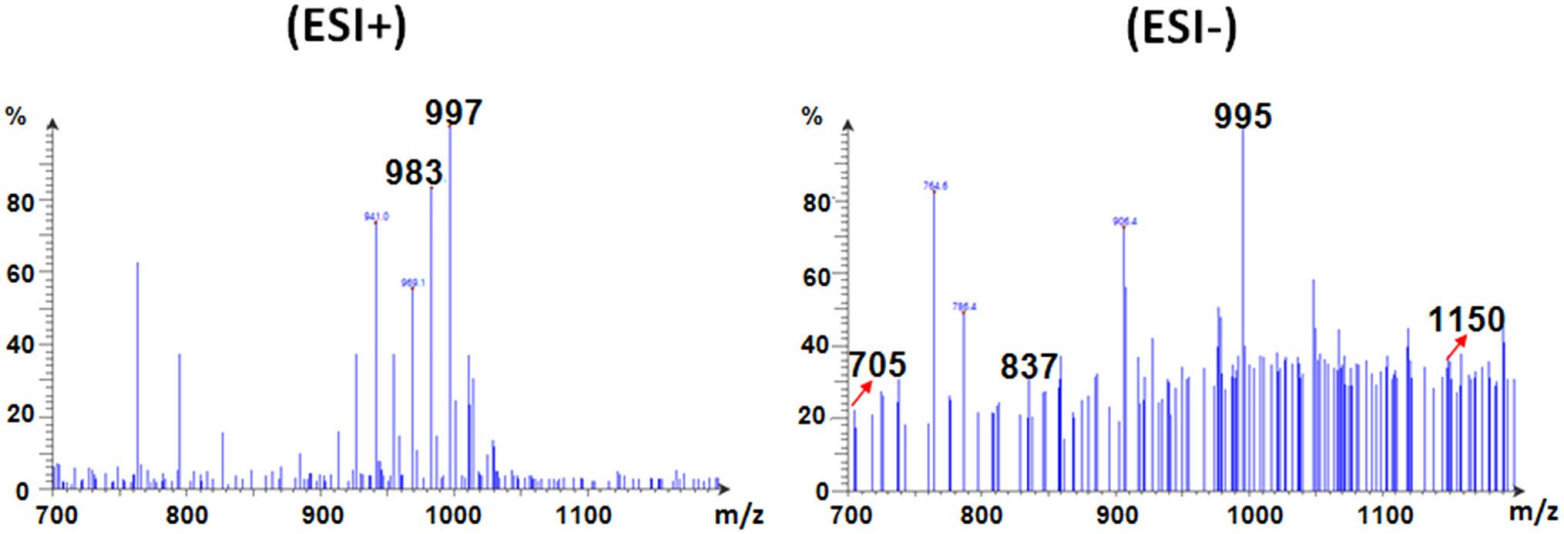
ESI-mass chart. Positive and negative ions ESI-mass chart recorded for GOS procured after 24 h

Thus, the various peaks presented in Fig. 9A could be used to verify the presence of GOS-3, 4, 5 and 6 after 3 h of reaction. Nonetheless, the ESI mode employed herein could induce fragmentation amid the GOS sample. Such ESI-induced fragmentation would be less intense than the fragmentations that would be induced if other carbohydrate ionization techniques, such as fast-atom-bombardment, were utilized (Kailemia et al. 2014). Accordingly, the intact GOS together with their constituting fragments could be represented as distinct peaks in the ESI mass charts. Hence, the peaks presented in Fig. 9A could be referred to the individual GOS 3, 4, 5, and 6 or to the intact GOS-6 and its fragments, especially that all the four aforementioned GOS were formerly reported to be concocted via the *A. oryzae* βGL (Sass and Jördening 2020; Todea et al. 2021; Yin et al. 2017). Upon prolonging the reaction duration beyond 3 h, peaks appeared ~ 1150 m/z (Figs. 9,10, and 11), especially in the ESI- charts. This might indicate the presence of GOS-7 (C_42_H_72_O_36,_ m.w. 1152). Analogously, the high DPN GOS (GOS-6) was formerly reported to be better presented in the ESI- charts than in the ESI + charts (Wahba 2021).

## Conclusions

The compromised stability of the SPI-GA-functionalized Car beads iβGL was resolved via simply soaking it with its substrate, lactose. The LT-iβGL presented superior thermal stability as was proved from its thermodynamic parameters especially its Δ*G* values, which were loftier than those presented by the free βGL at all temperatures. The storage stability of the LT-iβGL was also superior where 100.41 ± 1.08% relative activity was presented on the 43rd storage day. It could also be concluded that the post-immobilization uncontrolled enzyme-immobilizer interactions that occurred amid the iβGL and the SPI-GA-functionalized Car beads were mainly induced via physical interactions mediated via the immobilizer SPI entities.

## Data Availability

The author states that the data needed to reproduce the findings of this research are provided within the article.
